# Safety and efficacy of low dose naltrexone in a long covid cohort; an interventional pre-post study

**DOI:** 10.1016/j.bbih.2022.100485

**Published:** 2022-07-03

**Authors:** Brendan O'Kelly, Louise Vidal, Tina McHugh, James Woo, Gordana Avramovic, John S. Lambert

**Affiliations:** a Infectious Diseases Department, Mater Misericordiae University Hospital, Dublin 7, Ireland; b School of Medicine, University College Dublin, Dublin 4, Ireland

**Keywords:** SARS-CoV-2, COVID-19, Post COVID-19 syndrome, SF-12, Long covid

## Abstract

**Background:**

Up to 37.7% of patients experience symptoms beyond 12 weeks after infection with SARS-CoV-2. To date care for people with long covid has centred around multidisciplinary rehabilitation, self care and self pacing. No pharmacotherapy has been shown to be beneficial.

**Methods:**

In this single centre interventional pre post study, the safety of Low Dose Naltrexone (LDN) was explored in patients with Post COVID-19 Syndrome (PCS), defined by NICE as patients with ongoing symptoms 12 or more weeks after initial infections with SARS-CoV-2 where alternative explanation for symptoms cannot be found. Patients were recruited through a Post COVID clinic, had a baseline quality of life questionnaire in symmetrical Likert format, were prescribed 2 months (1 mg month one, 2 mg month two) of LDN and repeated the same questionnaire at the end of the second month. Patients were monitored to adverse events.

**Findings:**

In total 52 patients participated of whom 40(76.9%) were female. The median age was 43.5 years(IQR 33.2–49). Healthcare workers represented the largest occupational cohort n = 16(34.8%). The median time from diagnosis of COVID-19 until enrolment was 333 days (IQR 171–396.5). Thirty-eight participants (73.1%) were known to commence LDN, two of whom (5.3%) stopped taking LDN post commencement due to new onset diarrhoea and also described fatigue. In total 36(69.2%) participants completed the questionnaire at the end of the two-month period. Improvement was seen in 6 of 7 parameters measured; recovery from COVID-19, limitation in activities of daily living, energy levels, pain levels, levels of concentration and sleep disturbance (p ≤ 0.001), improvement in mood approached but was not significant (p = 0.054).

**Conclusions:**

LDN is safe in patients with PCS and may improve well-being and reduce symptomatology in this cohort. Randomised control trials are needed to further explore this.

## Highlights

• Low dose naltrexone (LDN) is safe to use in patients with long covid (LC).
• In patients with LC for a median 11 months, LDN reduced symptoms at 2 months.
• In this cohort, LDN also improved well-being in 6 of 7 parameters at 2 months.

## 1 Introduction

Currently with over 523 million infections worldwide (May 2022)(WHO, 2022), the COVID-19 pandemic continues to be a major medical, economic and social problem despite current vaccination efforts. Many patients will have persistence of symptoms beyond initial infection. The REACT-2 Study in the UK of over 78,000 community participants suggests up to 37.7% of symptoms can have symptoms beyond 3 months(Whitaker et al., 2021). Other studies report persistence of symptoms at 33–87% at 4 months(Nalbandian et al., 2021).

It remains unclear why some patients develop persistent symptoms of COVID-19, and interestingly this does not appear to be linked to severity of initial infection(Altmann and Boyton, 2021; Townsend et al., 2020, 2021). Reported symptoms include, but are not limited to, fatigue not relieved by rest, dizziness, headache, palpitations, chest pain, shortness of breath, fever, difficulty with concentration or thinking (“brain fog”), loss of sense of taste or smell, joint or muscle pain, gastrointestinal symptoms, depression and anxiety. Several hypotheses have been proposed to explain mechanisms for this post viral phenomenon including immune activation, endothelial damage and microthrombosis, neuroinflammation through microglial activation and brainstem dysfunction due to presence of angiotensin converting enzyme-2 and neuropilin-1 receptors have been proposed as mechanisms to explain neurological, psychological and autonomic sequelae(Crook et al., 2021; Yong, 2021). There does certainly appear to be objective evidence of multi-organ involvement in long-covid using magnetic resonance imaging at four months(Dennis et al., 2021). An explanation including a combination of peripheral, central, and psychological components may best explain these symptoms, more research is needed to better define this syndrome.

At present long covid clinics have been established in the UK and elsewhere(Care, 2021). To date there is no pharmacotherapy approved for long covid at present. A number of drug classes have been proposed as potential therapies including antihistamines and antidepressants. A Phase II pilot study (NCT04604704) exploring low dose naltrexone(LDN) and nicotinamide dinucleotide(NAD) effectiveness for symptoms of long covid is also underway.

Naltrexone is an opiate receptor antagonist at doses of 50 mg, but at lower doses of 1 mg–4.5 mg it appears to have unique immune modulation activity and is termed LDN. This may be due to antagonism of opioid growth factor receptor (OGFr), inhibition of toll-like receptor-4 inflammatory signalling, immunomodulation of macrophages and microglia, inhibition of T- and B-lymphocytes or other unknown mechanisms(Li et al., 2018). LDN has been shown to be beneficial for a number of conditions including crohn's disease; induction of remission and reduction in need for anti-inflammatory medications, chronic fatigue syndrome, reduction in use of disease modifying drugs in rheumatoid arthritis, fibromyalgia, multiple sclerosis and complex regional pain syndrome although studies are small (Bolton et al., 2020; Lie et al., 2018; Raknes et al., 2018; Raknes and Småbrekke, 2019; Younger et al., 2014).

In this safety study we explore the use of LDN in patients with long covid, who had symptoms for at least three months. A secondary aim of the study is to explore early signals of symptomatic improvement and improvement in well-being to support larger randomised control trial for the use of LDN for long covid.

## 2 Methods

### 2.1 Design

This is a prospective single centre interventional pre-post cohort study.

### 2.2 Setting

The study was done in the Mater Misericordiae University Hospital (MMUH), a 580 bed Tertiary Hospital and site of the National Isolation unit (NIU). Patients were recruited from the ‘COVID clinic’, created by the Infectious Diseases Directorate, between June and November 2020. A follow up clinic for all patients with a history of COVID-19. Patients followed in the clinic were those who had been hospitalised, those that were diagnosed in the emergency department and managed with home monitoring through the hospital, and referrals from general practitioners in the catchment area.

### 2.3 Eligibility

Patients were eligible if they had laboratory confirmed COVID-19, or in those with a clinical diagnosis of COVID-19 which requires individuals to have had symptoms of acute COVID-19 including fevers, SOB, cough, myalgia, anosmia or other symptoms in returning travellers or local patients at times where community transmission was known in those respective areas. Participants were required to be ≥ 18 years old, and to have any symptoms for at least six months after initial diagnosis of COVID-19 where other causes of symptoms had been excluded through appropriate investigations in the clinic, for example in obese patients with fatigue and high Epworth scores a work up may include anaemia work up, a sleep study, and thyroid function tests. Symptoms must not have preceded COVID-19 diagnosis and ultimately were deemed to be long covid.

### 2.4 Recruitment

Between 12 and 15 patients were seen in the clinic weekly, approximating 300 potential participants over the recruitment period. Baseline blood tests on initial clinic visit including full blood count, urea and electrolyte panel, liver blood tests, C-reactive protein, and up to date X-ray chest were done routinely. Additional tests including troponin, d-dimer, fibrinogen, echocardiography, spirometry or computerised tomography of the thorax, sleep studies were done if clinically indicated. At recruitment, a brief discussion about the rationale for use of LDN in the setting of patients with post COVID-19 syndrome (PCS). It was explained that the use of LDN in this context is not in line with current European Medicines Agency(EMA) recommendations and that there may be no improvement in symptoms. It was also explained that patients may experience adverse events and may need to stop LDN. The patients’ current medications were also reviewed prior to commencing LDN to explore any potential interactions.

### 2.5 Intervention

Patients who enrolled in the study received LDN 1 mg once daily for one month, the dose was increased by 1 mg monthly to a maximum of 3 mg. The aim was to see patients at a 2-month interval but due to contingency planning, staff in the clinic could be reallocated during new covid waves which would result in downscaling, service limitation and postponement of patient appointments. For this reason, three months of medication was prescribed and some patients who were seen after two months would have commenced a 3 mg dose for a number of days until their clinic appointment.

### 2.6 Outcomes

Patients commenced on LDN had assessment and questionnaires done at recruitment, and at two to three months. The questionnaire explored 7 different components of clinical status using Likert scaling, a symmetrically balanced ordinal scale assessment of each component; sleep(1–4), concentration(1–5), pain/discomfort(1–5), mood(1–5), energy levels(1–6), limitation in activities of daily living(1–3), and perception of overall recovery from COVID-19(1–5). Participants were also reviewed for adverse events and new symptoms at clinic reviews and LDN was stopped in this context if the patient had not already done so.

### 2.7 Data

Data was pseudo-anonymised and collected using Microsoft Excel 2019®. The data was password protected and stored on hospital firewall protected hospital server and was only accessed by members of the research team.

### 2.8 Statistical analysis

Statistical analysis was done using IBM SPSS V24.0 (IBM Corporation, Armonk, NY, USA). Categorical data are presented as number, percentages and 95% Confidence intervals(CI). Numeric and ordinal data are presented as median and interquartile range (IQR). Normality testing was done using Shapiro wilks tests. Normally and non-normally distributed paired data were analysed using paired T-test and Wilcoxon signed rank test respectively. Effect size was calculated by Rosenthal correlation coefficient. Chi squared test or Fisher's exact test were used to compare categorical data. A p value of <0.05 was deemed significant for individual statistical tests.

## 3 Results

In total 52 patients participated of whom 40(76.9%, CI 65.4–88%) were female. The median age was 43.5 years(IQR 33.2–49). The most common comorbidities were hypertension n = 5(9.6%, CI 0–17.6%) and dyslipidaemia n = 5(9.6%, CI 0–17.6%). Healthcare workers represented the largest occupational cohort n = 16(34.8%, CI 21.4–48%) and 12 (23%, CI 11.6–34.5%) patients were admitted during initial illness, Table 1.

**Table 1:** Patient demographics.

Demographics, co-morbidities and level of care during acute COVID illness	N(%)	95CI%
Total	52	
Median age in years (IQR)	43.5(33.2–49)	
Female	40(76.9)	65.4–88
**Occupation**		
Healthcare worker	16(30.8)	18.2–43.3
Other	30(57.7)	44.3–71.1
Retired/unemployed	4(7.7)	0–14.9
None documented	2(3.8)	0–9
**Co-morbidities**		
Hypertension	5(9.6)	0–17.6
Dyslipidaemia	5(9.6)	0–17.6
Obesity	2(3.8)	0–9
Diabetes mellitus	2(3.8)	0–9
Mental Health	7(13.5)	4.2–22.7
Asthma	9(17.3)	7–27.6
Nil reported	4(7.69)	0–14.9
Maximum level of care*		
Hospital admission	12(23.1)	11.6–34.5
High dependency unit admission	2(3.8)	0–9
Community monitoring	38(73.1)	61–85.1

*Maximum necessary level of care due to disease severity during acute presentation with COVID-19.

Baseline investigations for those enrolled were done to explore symptoms to explore alternative diagnoses. Three patients had anaemia (Hb 10.9, 10.4, 11.2 mg/dL), two of whom had shortness of breath and fatigue, although other symptoms were found in these patients not explained by anaemia. No patients had neutrophilia, CRP was raised in five patients (range 10–24 mg/L), creatinine is raised in two patient 92,94μmol/L, alanine aminotransferase was raised in 5 (range 59–140 IU/L). Of those that had additional tests 3/18 d-dimers were raised(range 0.61–1.09 mg/L), 0/18 troponin levels were raised, ferritin was raised in 5/28 (range 313-609μg/L), and fibrinogen was within reference range for 0/14. Of 41 patients that had X-ray chest imaging done, no concerning changes were identified apart from bi-apical pleural scarring in a single patient. Transthoracic echocardiogram was done in 17 patients, no ventricular dysfunction, myocarditis or pericarditis was seen. 24 h Holter monitor was done in 12 patients, occasional premature ventricular contractions were seen in three patients and mild airflow limitation and scalloping of flow volume loop was seen in two of 12 pulmonary function tests performed, the remaining showed no concerning abnormalities.

The median time from diagnosis of COVID-19 until enrolment was 333 days(IQR 171–396.5).The most common symptoms at the time of enrolment were fatigue n = 47(90.4%), low mood n = 40(76.9%) headache n = 39(75%), brain fog n = 39(75%), sleep disturbance n = 37(71.2%), shortness of breath n = 36(69.2%) dysthesia n = 29(55.8%), anxiety n = 29(55.8%), palpitations n = 31(59.6%), chest pain/tightness 28(53.8%), tinnitus n = 24 (46%), dizziness n = 24(46%), changes to taste or smell n = 23(44.2%) and cough n = 19(36.5%).

**Fig. 1:**
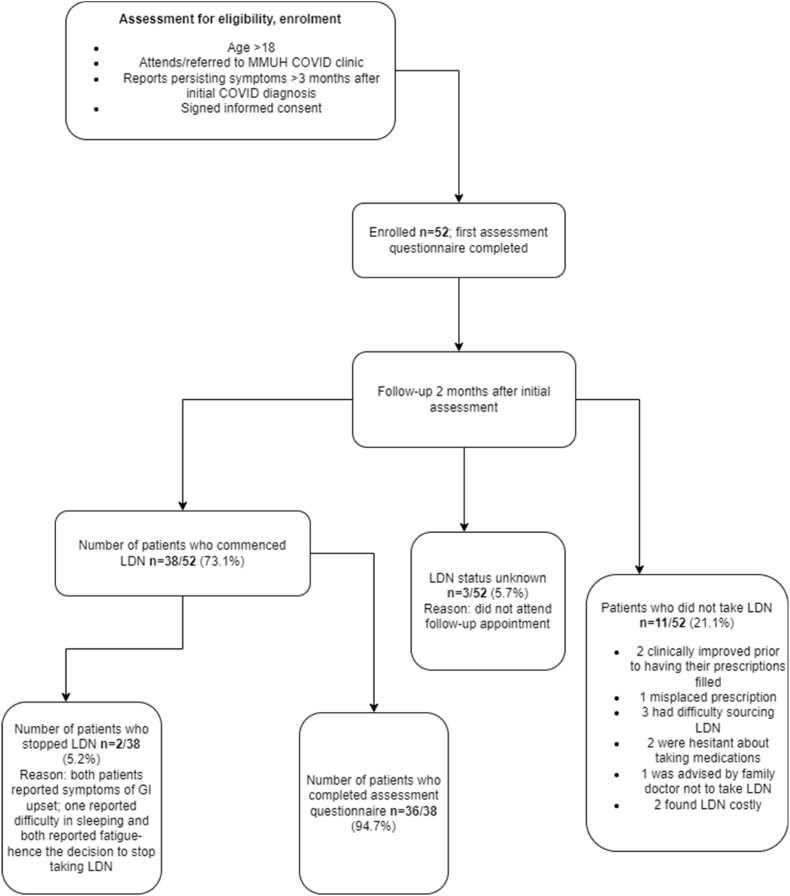
Flow diagram of those enrolled, retained and lost to follow up.

After initial enrolment, 38 patients commenced LDN and in total 36 (69.2%, CI 56.6–81.7%) completed a follow up questionnaire at a median 63 days(IQR 56–84). Adverse events in two patients (diarrhoea and fatigue in both) resulted in discontinuation prior to the second questionnaire. There were several reasons for non-commencement some of which can be contributed to LDNs off label use including cost and inability to access the drug on the Irish Drug Payment Scheme, also sourcing the drug and hesitancy amongst others. Reasons for discontinuation of LDN can be seen in Fig. 1. Of 50 patients in employment, 31(64.6%, CI 51–78%) had returned to work yet 34(70.8%, CI 58–83.6%) report either not being able to return to work or having significant disruption to work due to symptoms post COVID-19.

At follow up there was improvement in 6 of 7 parameters measures using symmetrical Likert scales; recovery from COVID-19, limitation in activities of daily living, energy levels, pain levels, levels of concentration and sleep disturbance, Table 2. The largest effect was seen in levels of pain (Effect size −0.534). Mood was not significantly reduced at 8 weeks on a five point likert scale (p = 0.054). Overall there was reduction in reporting of individual symptoms for all symptoms with a significant reduction (p < 0.05) seen in low mood, personality change, joint pain, chest tightness and cough, and a trend (p < 0.10) towards significance for; fatigue, shortness of breath, brain fog, sleep disturbance, and dysthesia, Table 3.

**Table 2:** Questionnaires at baseline and 2 month follow up.

Likert scale	Baseline questionnaire median(IQR)	2 month follow up median(IQR)	P value (2 tailed <0.05)	Z score (based on negative ranks)	Effect size (Rosenthal coefficient)
	N = 36	N = 36			
I feel I have recovered from COVID-19 (1–5)	1.5(1–2)	2(2–4)	**<0.001**	−4.492	−0.515
Does you health now limit you in you in daily activities? How much (1–3)	1(1–2)	2(1–2)	**0.001**	−3.207	−0.368
In the past 4 weeks do you have a lot of energy? (1–6)	3(2–3)	3(3–4)	**0.001**	−3.334	−0.382
In the past 4 weeks rate your overall mood(1–5)	2(2–3)	3(2–3)	.054	−1.925	−0.221
In the past 4 weeks rate you pain/discomfort(1–5)	2(2–3)	4(3–4)	**<0.001**	−4.66	−0.534
In the past 4 weeks rate your level of concentration(1–5)	2(1–2)	2(2–3)	**0.001**	−3.337	−0.382
Have you trouble staying or falling asleep(1–4)	2(1–3)	3(1–3)	**<0.001**	−3.896	−0.447

**Table 3:** Incidence of reported symptoms at baseline and at 2 months.

Symptoms	Baseline n(%)	2 month follow up n(%)	P value (2 tailed <0.05)
Total	36	36	
Fatigue	33(91.7)	27(75)	.085
Fevers	6(16.7)	2(5.6)	.185
Sore throat	13(36.1)	8(22.2)	.136
Anosmia/dysgeusia	16(44.4)	11(30.6)	.171
Hair loss	11(30.6)	9(25)	.65
Tinnitus	17(47.2)	12(33.3)	.2
Chest pain/tightness	20(55.6)	12(33.3)	**.047**
Palpitations	22(61.1)	16(44.4)	.132
Cough	19(37.3)	5(13.9)	**.016**
Shortness of breath	25(69.4)	19(52.8)	.09
Headache	27(75)	24(63.9)	.314
Dizziness	17(47.2)	14(38.9)	.618
Brain fog	27(75)	20(55.6)	.072
Sleep disturbance	26(72.2)	16(44.4)	.058
Dysthesia	20(55.6)	13(36.1)	.056
Abdominal discomfort/bloating	17(47.2)	14(38.9)	.449
Nausea/Vomiting	12(33.3)	6(16.7)	.083
Diarrhoea	14(38.9)	9(25)	.166
Joint pain	26(72.2)	13(36.1)	**.008**
Myalgia	20(55.6)	14(38.9)	.163
Low mood	28(77.8)	17(47.2)	**.003**
Anxiety	20(55.6)	16(44.4)	.337
Personality change	9(25)	0(0)	**.001**

## 4 Discussion

In this study patients who were symptomatic for a median 333 days received LDN over a two-three month period(median 63 days, IQR56-84) showed improvement in self-reported limitation in activities of daily living, energy levels, pain levels, levels of concentration, sleep disturbance and overall recovery from COVID-19 using a Likert scale questionnaire. We also noted a reduction in reporting of individual symptoms including joint pain, low mood, personality change, chest tightness and cough. Although there appears to be a signal for improvement in symptoms it is not possible to solely attribute this to LDN due to the limitations of the study, primarily the lack of a control arm. Interestingly the largest effect in improvement was in pain, LDN has been shown to alleviate chronic pain in a number of studies(Younger et al., 2014). The improvements in pain amongst nearly all other parameters suggests that there may predominately be a central nervous system mediated aetiology in the symptomatology of long covid and that taking LDN which has some activity on glial cells may be beneficial for a number of symptoms.

Two patient had document adverse effects which resulted in cessation of therapy, indicating safety in 94.7% in those that took LDN as prescribed until follow up. Albeit three additional patients were lost to follow and it is unknown if they took LDN. LDN has been previously shown to have a high safety profile. In one study of MS patients, 77% report no adverse effects, no patients were hospitalised, and none had drug-drug interactions with disease modifying drugs(Turel et al., 2015). Overall LDN has been demonstrated to be very safe with no significant interactions with primary treatments for immunological disorders or pain medications(Patten et al., 2018).

Approaching long covid in a similar way to other conditions like chronic fatigue syndrome or myalgic encephalomyelitis with graded exercise therapy and cognitive behavioural therapy may be beneficial but is not supported by evidence(Crook et al., 2021). The National Institute for Health and Care Excellence(NICE) have recently published recommendations on the management of long covid that highlight the lack of evidence for rehabilitation or pharmacotherapy, they promote self-management including self-pacing and caution the use of medications for short term gain that may have long term adverse effects(NICE, 2021). The panel did reach consensus on the potential benefits of multidisciplinary and patient centred approach to rehabilitation after appropriate investigation for alternative diagnoses that could explain symptoms. Although LDN in this study and from previous studies has been shown to be a very safe medication, even if proven to be efficacious for long covid with randomised controlled trials, a niche for its use will need to be defined; which patients it should be used in and how long it should be continued for.

Furthermore, long covid at present is described broadly as two syndromes dependent on duration of symptoms and absence of an alternative explanation for symptoms, described by NICE as *on-going symptomatic COVID-*19 for people who still have symptoms between 4 and 12 weeks after initial symptoms, and *post-COVID 19 syndrome* in those with symptoms or more than 12 weeks. These definitions may change over time as more research sheds light on the mechanisms that underly the persistence of symptoms a spectrum of conditions may be defined. LDN may be a nuanced treatment for some patients with certain symptomatologic phenotypes in this scenario.

This study has several limitations; it was done in a single centre and has a small sample size. No control arm was used in the study, only pre and post analysis was done and the witnessed improvements in symptoms and wellbeing may be due in part of fully to placebo or natural improvement in symptoms. Patients were provided with a prescription for LDN and were required to source this and pay for it themselves, this may account for the failure to retain a number of patients, also formulations of LDN were not standardised by this methodology. Also, no objective parameters of improvement were recorded (i.e. heart rate etc) only self-reporting through repeat questionnaires in a follow up clinic. The study is also of relatively short duration at two to three months.

In summary larger more robust studies are needed to explore the safety and efficacy of LDN in long covid patients. In this study it appears LDN is quite safe and may be beneficial in alleviating a number of symptoms and improving function over a relatively short time period.

## Ethical statement

This study was approved by the Mater Misericordiae University Hospital ethics committee. Institutional Review Board Reference: 1/378/2141.

## Consent for publication

Not applicable.

## Patient and public involvement

Neither patients nor the public were involved in the design or conduct of the study, the choice of outcome measures, or recruitment of the study. The results of the study were presented to patients and the public at a formal Patient and Public Involvement event.

## Availability of data

Data will be made available upon request.

## Funding

This work was supported by the 10.13039/100010414Health Research Board (HRB), [COV19-2020-123].

## Declaration of competing interest

The authors declare the following financial interests/personal relationships which may be considered as potential competing interests: Brendan O’Kelly reports financial support was provided by UCD Foundation (Gilead, Pfizer, GSK Newman Fellowship). John S Lambert reports financial support was provided by Health Research Board Ireland.
